# Subliminal versus supraliminal stimuli activate neural responses in anterior cingulate cortex, fusiform gyrus and insula: a meta-analysis of fMRI studies

**DOI:** 10.1186/s40359-014-0052-1

**Published:** 2014-12-11

**Authors:** Paolo Meneguzzo, Manos Tsakiris, Helgi B Schioth, Dan J Stein, Samantha J Brooks

**Affiliations:** Department of Psychiatry and Mental Health, University of Cape Town, Anzio Road, Cape Town, 7995 South Africa; Department of Neuroscience, University of Padua, Padova, Italy; Lab of Action and Body, Department of Psychology, Royal Holloway, University of London, London, UK; Department of Neuroscience, Uppsala University, Uppsala, Sweden

**Keywords:** Subliminal, Supraliminal, Activation Likelihood Estimation, ANterior cingulate cortex, Fusiform gyrus, Cingulate cortex, Insula

## Abstract

**Background:**

Non-conscious neural activation may underlie various psychological functions in health and disorder. However, the neural substrates of non-conscious processing have not been entirely elucidated. Examining the differential effects of arousing stimuli that are consciously, versus unconsciously perceived will improve our knowledge of neural circuitry involved in non-conscious perception. Here we conduct preliminary analyses of neural activation in studies that have used both subliminal and supraliminal presentation of the same stimulus.

**Methods:**

We use Activation Likelihood Estimation (ALE) to examine functional Magnetic Resonance Imaging (fMRI) studies that uniquely present the same stimuli subliminally and supraliminally to healthy participants during functional magnetic resonance imaging (fMRI). We included a total of 193 foci from 9 studies representing subliminal stimulation and 315 foci from 10 studies representing supraliminal stimulation.

**Results:**

The anterior cingulate cortex is significantly activated during both subliminal and supraliminal stimulus presentation. Subliminal stimuli are linked to significantly increased activation in the right fusiform gyrus and right insula. Supraliminal stimuli show significantly increased activation in the left rostral anterior cingulate.

**Conclusions:**

Non-conscious processing of arousing stimuli may involve primary visual areas and may also recruit the insula, a brain area involved in eventual interoceptive awareness. The anterior cingulate is perhaps a key brain region for the integration of conscious and non-conscious processing. These preliminary data provide candidate brain regions for further study in to the neural correlates of conscious experience.

**Electronic supplementary material:**

The online version of this article (doi:10.1186/s40359-014-0052-1) contains supplementary material, which is available to authorized users.

## Background

Recent brain imaging evidence suggests that subliminal stimuli can alter behavior, via non-conscious processes (Muscarella et al. 2013; Eimer & Schlaghecken 2003). Neural models of behavior elicited by non-conscious stimuli implicate the prefrontal and cingulate cortices in the regulation of subcortical brain regions linked to impulsive and largely non-conscious stimulus perception (Ochsner et al. 2012). In this way therefore, one might suggest that conscious cognitive processes, such as decision-making and working memory that are associated with prefrontal cortex networks, are influenced by non-conscious experiences. William James and Carl Lange, who were the first to provide theories for non-conscious processes in the decision making capabilities of the human mind, postulated the importance of physiological mechanisms that are not at first consciously perceived, e.g. that physiological changes in the body following an event lead to a response that drives one’s conscious decision-making processes (Cannon, 1927). Some of James and Lang’s views are in line with contemporary notions of the unconscious mind, and some of these theories are beginning to be reflected in neuroimaging studies (19, 21, 52).

Processing of non-conscious physiological responses in the body by the cortex is a view that has been incorporated into many contemporary theories. One example by Damasio (54) and Tranel (55) proposes that emotions, which help us to make decisions, are cognitive stories constructed by the cortex in a particular context to explain bodily arousal; a view reflected in their recently updated Somatic Marker Hypothesis, highlighting the importance of brainstem (e.g. the periaquaductal gray) activation in conscious experience (Damasio 2010; Panksepp 2011). Perception of heart rate variability, a largely automatic physiological process, can also influence the modulation of cognitions and emotions (Kim et al. 2013). Others suggest overlapping but different neural circuitry in consciousness, incorporating brain processing in both non-conscious subcortical and conscious prefrontal regions respectively (Ochsner et al. 2012). Against this background, non-consciously perceived stimuli we hypothesise, should therefore activate different brain regions to stimuli that are consciously perceived.

A recent qualitative review of subliminal findings in functional Magnetic Resonance Imaging (fMRI) research reports that non-consciously perceived stimuli can influence perceptual, lexical and semantic processing, but that the neural response to subliminal stimuli depends on the strength of stimulus presentation, as well as individual differences in threshold for conscious perception (Kouider & Dehaene 2007). Furthermore, this review distinguishes between subliminal and preconscious awareness, which may be reflected in varying degrees of cortical versus subcortical recruitment, although the various paradigms used to measure this limit the conclusions. Another recent review revealed that the non-conscious processing of motor responses involves the precuneus and supplementary motor areas, whereas subjective experience of voluntary action may involve fronto-parietal network activation (D'Ostilio & Garraux 2012). A recent review of electrophysiological evidence of brain function shows that error detection, a psychological function often associated with the anterior cingulate cortex (ACC) occurs non-consciously (Shalgi & Deouell 2013). Thus, there is now ample neurobiological evidence to suggest that conscious and unconscious processing may have some overlap, but that the origins may occur independently and in specific brain areas. However, there has been no meta-analysis of fMRI studies that measure different degrees of conscious perception using the same stimulus.

Subliminal neuroimaging paradigms using masked and thus non-consciously perceived stimuli provide a potential method to progress knowledge of the neural correlates of non-conscious, primary processes in the brain. For example, a recent meta-analysis of functional fMRI studies has shown that subliminal arousing (versus subliminal neutral) stimuli evoke distinct activations in primary visual areas, somatosensory regions, implicit memory and conflict monitoring systems independent of conscious awareness of the stimulus (Brooks et al. 2012). This large meta-analysis demonstrated a distinct lack of prefrontal cortex activation in response to non-consciously perceived stimuli. However, this review did not explicitly analyze differential neural activation to the same conscious, versus - unconsciously perceived arousing stimuli, which would go some way to delineate which regions are involved in conscious processing. While there is variability in fMRI methods, in terms of the contrasts applied, participants studied, stimulus presentation employed, coordinate systems adopted (e.g. MNI, Talairach, AFNI), statistical analyses used, a basic meta-analysis of fMRI data can yield useful data with Activation Likelihood Estimation (ALE) (Laird et al. 2005; Eickhoff et al. 2009; Eickhoff et al. 2010; Turkeltaub et al. 2011). ALE is a method that is currently being used extensively in the neuroimaging field. However, no meta-analysis has yet examined differential neural activation in fMRI studies measuring conscious (supraliminal) versus unconscious (subliminal) perception of the same stimulus. By doing so, we might provide a preliminary delineation of activated brain regions associated with conscious versus non-conscious perception, to guide further studies in the field.

Here, we are the first to conduct an exploratory analysis of brain regions in healthy subjects that are activated to subliminal and supraliminal stimuli. We use the ALE approach to meta-analyse fMRI studies reporting neural activation in response to both the subliminal and supraliminal presentation of the same stimulus. Specifically, we meta-analyse only those fMRI study publications that used the same stimuli (but at different perception thresholds) with the same participants and the same experimental conditions within the same publication. In all studies included, subliminal perception was confirmed by a forced choice task. This meta-analysis differs from our recently published meta-analysis where only fMRI studies using subliminal stimuli (arousing versus neutral) were included with no activation to supraliminal perception (Brooks et al. 2012).

By contrast, this meta-analysis attempts to answer a different question: how does conscious cognitive modulation of a stimulus, relative to the same stimulus being perceived unconsciously, alter brain activation? By illustrating here the core clusters of neural activation across studies that contrast the level of subjective awareness of a stimulus, we aim to delineate the regions associated with conscious experience from regional activation associated with stimulus perception that is not at first consciously experienced. In line with contemporary theories and our recent meta-analyses, we hypothesise that consciously perceived stimuli will activate prefrontal and anterior cingulate cortex regions linked to conscious cognitive evaluation, whereas the same unconsciously perceived stimuli will provoke relatively greater activation in subcortical brain regions linked to implicit memory and arousal, such as the hippocampus, amygdala, striatum and primary visual cortex.

## Methods

### Searching

#### Inclusion and exclusion criteria

PubMed, Medline, Ovid, Sciencedirect, Web of Science and Google Scholar were searched, and hand searches of reference lists up to October 2013. Search terms for online searches included fMRI and MRI, with subliminal and supraliminal stimulation as our search criteria. To be included in our meta-analysis, studies met the following criteria: a) studies were published within the last decade, between January 2001 to October 2013, b) published in a peer-reviewed journal, c) used a task that utilized both the subliminal and supraliminal presentation of the *same* arousing stimulus, c) the study included a direct contrast between brain activation to subliminal and supraliminal stimulus presentation, d) were original articles written in English, e) used functional Magnetic Resonance Imaging (fMRI) and not other brain imaging modalities (e.g. Positron Emission Tomography, [PET], Transcranial Magnetic Stimulation [TMS]) so that the data could be better aggregated for meta-analysis, and f) reported the neural activation coordinates in Montreal Neurological Institute (MNI) or Talairach space (Talairach & Tournoux 1988). Studies examining people with physiological conditions who were without a psychiatric comorbid diagnosis were included (Irritable Bowel Syndrome, IBS, Gastro-esophageal reflux disease, GERD). We excluded otherwise eligible fMRI studies that only used Region of Interest (ROI) analysis as there is robust evidence that these studies artificially inflate ALE analyses (Eickhoff et al. 2009). Study selection was done by three researchers (PM, SJB and HBS) and cross-checked between them. For a list of excluded studies, see Additional file 1: Table S1. For details of our meta-analysis MOOSE checklist inclusions, see Additional file 2: Table S2.

#### Selected studies

We found 77 studies that were initially screened for inclusion in the systematic review, but 20 of these did not meet the eligibility criteria described above. Of these 57 eligible studies, 13 were not included in the meta-analyses because they did not provide details of Talairach or MNI peak activation coordinates, and we were not able to contact the authors. Of the 44 fMRI studies to date, only 16 of these explicitly analyzed contrasts between subliminal and supraliminal thresholds of the same arousing stimulus or analyzed subliminal/supraliminal stimulation with a methodology similar to the one used in studies implying direct comparison between two different kinds of stimulation (the other studies compared only subliminal neutral vs. subliminal arousing stimuli). Of the 16 remaining studies with subliminal vs. supraliminal studies with some overlap in studies presenting both subliminal and supraliminal stimuli, 6 of these were excluded because they used exclusively Region of Interest (ROI) analysis, a technique that analyzes only a small region of the brain, based on a priori hypotheses. This is in contrast to a Whole Brain (WB) analysis, which statistically analyzes activation across the whole brain in one analysis. Thus, this left 10 WB fMRI studies that specifically included brain imaging coordinates for both subliminal and supraliminal perception, uniquely, of the same affective stimulus. It must be noted that one of the 10 studies directly compared subliminal with supraliminal presentation of the same stimulus, but only reported differential activation in the supraliminal condition, resulting in 9 studies contributing to the subliminal condition, and 10 studies contributing to the supraliminal condition. We included studies that either provided a direct comparison between subliminal versus supraliminal stimulation, or compared against a neutral condition (thus biasing the activation reported towards either subliminal or supraliminal perception). See Table 1 for a list of included studies.

**Table 1:** **List of studies included in the ALE meta-analyses**

**Study name**	**Type of subject, gender, mean age**	**Subliminal condition**	**Supraliminal condition**	**n**	**Foci**	**fMRI analysis**	**Activation threshold**
**a) Subliminal activation greater than supraliminal activation**							
Diekhof et al. 2009	Healthy: 4 male, 5 female, 24.4 years (S.D. 2.2)	Subtle changes in audio frequency	Detectable changes in audio frequency	9	1	WB_e_	p < 0.001
Lawal et al. 2006	Irritable Bowel Syndrome: 10 female, Healthy: 10 female. 19–38 years	Rectal stimulation (bag inflated below perception threshold)	Rectal stimulation (bag inflated above perception threshold)	18	17	WB_b_	p < 0.05
Phillips et al. 2004	Healthy: 5 males (grp.1), 29.5 years (S.D. 4.7) 5 male (grp. 2) 28.4 years (S.D. 6.2)	Covert angry and disgusted faces	Overt angry and disgusted faces	8	23	WB_b_	p < 0.005
Prochnow et al. 2013	Healthy: 5 men, 7 women, 23.8 years (S.D. 3.0)	Covert facial expressions of happiness, anger, sadness	Overt facial expressions of happiness, anger, sadness	12	11	WB_e_	p < 0.05 (2 foci at p < 0.01)
Sidhu et al. 2004	Irritable Bowel Syndrome: 8 female, Healthy: 8 female, 19–38 years	Rectal stimulation (bag inflated below perception)	Rectal stimulation (bag inflated at perception and above perception)	16	64	WB_b_	p < 0.05
**Only subliminal stimulation**							
Duan et al. 2010	Healthy: 5 males, 13 females, 23.6 years (S.D. 1.3)	Covert surprised faces		18	41	WB_b_	p < 0.001
Kouider et al. 2009	Healthy: 8 males, 8 female, 23 years (S.D. 2)	Covert famous faces		16	9	WB_e_	p < 0.001
Smith et al. 2011	Healthy female, 29 years (range 19–53)	Rectal stimulation (bag inflated below perception)		14	13	WB_b_	p < 0.001
Song et al. 2006	Irritable Bowel Syndrome: 12 female, Healthy: 12 female. 23 years (S.D. 0.3/S.D. 0.92)	Rectal stimulation (bag inflated below perception)		24	13	WB_b_	p < 0.001
				135	192		
**b) Supraliminal activation greater than subliminal activation**							
Diekhof et al. 2009	Healthy: 4 male, 5 female, 24.4 years (S.D. 2.2)	Subtle changes in audio frequency	Changes in audio frequency	9	27	WB_e_	p < 0.001
Gillath & Canterberry, 2011	Healthy: 19 male. 20 female, 19.65 years (no avail S.D.)	Masked sexual images presented at 23 ms	Supraliminal masked sexual images presented at 524 ms	39	21	WB_e_	p < 0.001
Lawal et al. 2006	Irritable Bowel Syndrome: 10 female, Healthy: 10 female. 19–38 years	Rectal stimulation (bag inflated below perception threshold)	Rectal stimulation (bag inflated above perception threshold)	18	25	WB_b_	p < 0.05
Phillips et al. 2004	Healthy: 5 male (grp.1), 29.5 years (S.D. 4.7) 5 male (grp. 2) 28.4 years (S.D. 6.2)	Covert angry and disgusted faces	Overt angry and disgusted faces	8	32	WB_b_	p < 0.005
Prochnow et al. 2013	Healthy: 5 men, 7 women, 23.8 years (S.D. 3.0)	Covert facial expressions of happiness, anger, sadness	Overt facial expressions of happiness, anger, sadness	12	11	WB_e_	p < 0.05 (2 foci at p < 0.01)
Sidhu et al. 2004	Irritable Bowel Syndrome: 8 female, Healthy: 8 female, 19–38 years	Rectal stimulation (bag inflated below perception)	Rectal stimulation (bag inflated at perception and above perception)	16	136	WB_b_	p < 0.05
**Only supraliminal stimulation**							
Hall et al. 2010	Irritable Bowel Syndrome: 7 female, Healthy: 6 female, 30–40 years		Rectal stimulation (bag inflated above pain perception)	13	26	WB_e_	p < 0.001
Kouider et al. 2009	Healthy: 8 males, 8 female, 23 years (S.D. 2)		Overt famous faces	16	8	WB_e_	p < 0.001
Smith et al. 2011	Healthy female, 29 years (range 19–53)		Rectal stimulation (bag inflated above pain perception)	14	9	WB_b_	p < 0.001
Song et al. 2006	Irritable Bowel Syndrome: 12 female, Healthy: 12 female. 23 years (S.D. 0.3/S.D. 0.92)		Rectal stimulation (bag inflated above pain perception)	24	20	WB_b_	p < 0.001
				169	315		
			***Grand Total:***	**304**	**507**		

_b_Block design fMRI, _e_Event-related fMRI, n = number of participants, foci = number of separate Talairach coordinates contributing to the meta-analysis, n = number of participants, WB = Whole Brain Analysis, S.D. = Standard deviation. Note: all participants had no psychiatric comorbidities.

#### Definition of subliminal and supraliminal stimuli

FMRI studies included in this meta-analysis contrast neural activation to subliminal and supraliminal presentation of the same stimuli (see Table 1 for details of the contrast for each study). Contemporary definitions of subliminal stimulation purport that stimuli are rendered subliminal if the stimuli are not perceived consciously by the participant (20,31,32). Subliminal stimulation is, in comparison to consciously-perceived stimulation, relatively weak and of low-intensity, suggesting that the neural processes driving unconsciously-perceived stimuli are less sophisticated and at the lower-order of function (Bargh & Morsella 2008). The effects of subliminal stimuli can now be measured in brain imaging studies, examining the brain processes involved. Subliminal stimulation is not accessible to conscious introspection, which means that the presentation of such stimuli cannot be consciously recollected (Shalgi & Deouell 2013). Subliminal presentation is most often achieved by a brief stimulus onset asynchrony (SOA) usually not more than 50 ms, followed by a ‘masking’ procedure. Backward masking is the most common, where another stimulus is presented directly after the subliminal stimulus, preventing conscious perception (Breitmeyer et al. 2007). In the present search, all studies included in the review presented the same stimulus both at a subliminal and a supraliminal level (see Table 1).

#### Quantitative data synthesis: ALE meta-analyses

**Figure 1:**
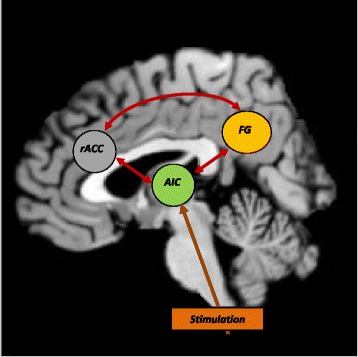
**Topographical representation of the brain regions illustrated by the meta-analysis.** ACC: Anterior Cingulate Cortex; r: rostral; c: caudate; FG: fusiform gyrus. To illustrate a topographical representation of our results we used the Colin27_T1_seg_MNI template provided on the GingerALE website (http://www.brainmap.org).

To examine relative activation to the subliminal and supraliminal presentation of arousing stimuli, we conducted two separate meta-analyses using BrainMap GingerALE version 2.3.1 software (Laird et al. 2005). The ALE method is a voxel-wise technique which provides information from convergence in the spatial location of neural correlates across studies. Neural correlates, or foci from included studies become “activation likelihoods” for each voxel in the brain and, for each one ALE gives a score using a three-dimensional Gaussian probability density function doing an estimation considering also the number of subjects in each study. The Gaussian distributions are then summed across studies to generate a map that estimate the likelihood of activation for each voxel (Laird et al. 2011; Turkeltaub et al. 2012). We applied the updated version of the ALE approach (Eickhoff et al. 2010) to conduct the meta-analyses using Talairach coordinates (“foci”) from neuroimaging results and converting Montreal Neurological Institute (MNI) coordinates in to Talairach for the analysis using GingerALE software. As suggested by Eickhoff et al. in their technical note (28) we used a threshold of p < 0.05 with cluster-level corrected inference using p < 0.001 uncorrected at voxel-level as the cluster-forming threshold. This was to ensure that only highly significant clusters were reported. We used an anatomical image overlay program called Mango (Creators, Jack Lancaster, Michael Martinez: http://ric.uthscsa.edu/mango) to illustrate the results of our meta-analyses, using the Colin27_T1_seg_MNI template provided on the GingerALE website (http://www.brainmap.org). We also used the Colin27_T1_seg_MNI template to produce the schematic summary of our findings in Figure 1.

## Results

### Meta-analysis one: subliminal stimulation > supraliminal stimulation

From 192 foci, 154 subjects and 9 separate experiments, 3 significant clusters were found that survived the cluster level inference threshold. Cluster one was found in right fusiform gyrus/middle occipital gyrus (x = 47, y = −71, z = −3) in BA 19, cluster two was found in right caudal anterior cingulate cortex (x = 2, y = 32, z = 36) in BA 32 and cluster three was found in right insula (x = 37, y = 4, z = −5) in BA 13.

### Meta-analysis two: supraliminal stimulation > subliminal stimulation

From 320 foci, 188 subjects and 10 separate experiments, 2 significant clusters were found that survived the cluster level inference threshold. Cluster one was found in left anterior cingulate cortex (x = −2, y = 34, z = 18) in BA 32, cluster two was found in mid-caudal anterior cingulate cortex (x = 0, y = 19, z = 31) in BA 32.

**Figure 2:**
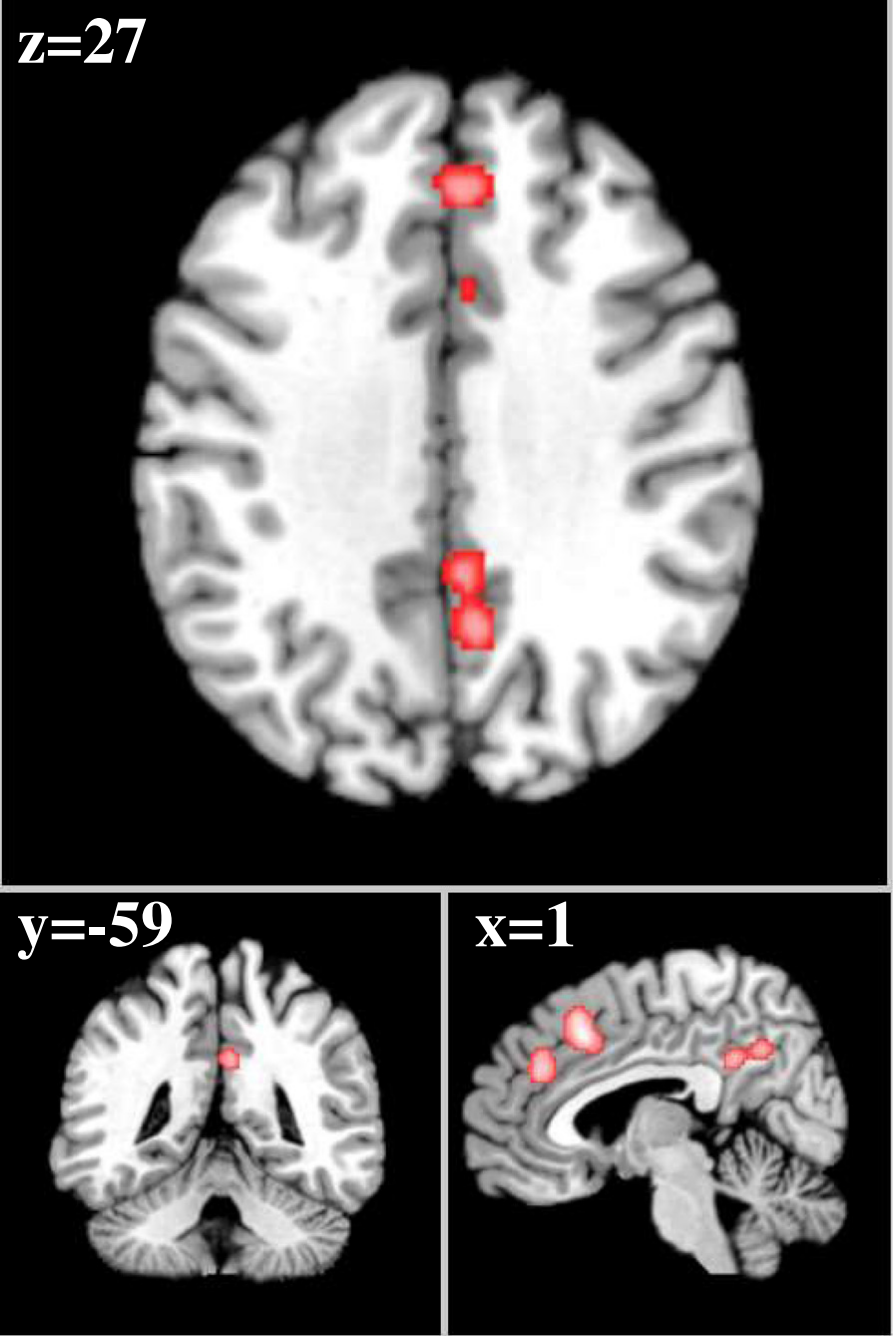
**Right fusiform gyrus (subliminal).** ALE significant activation to subliminal > supraliminal arousing stimuli in Brodmann Area 19, with a cluster size of 1008 voxels mm^2^, ALE value = 4.12.

**Figure 3:**
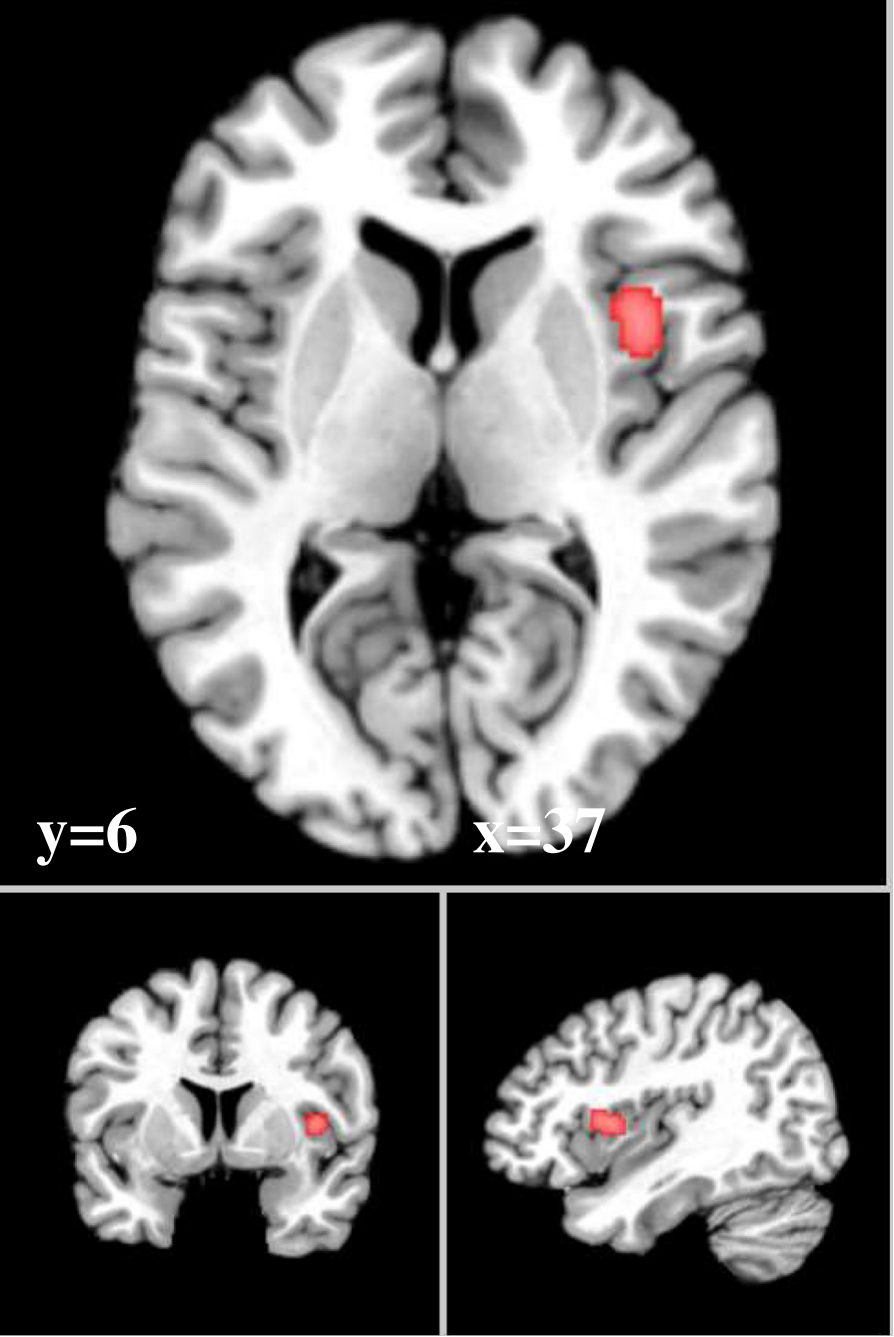
**Right insula (subliminal).** ALE significant activation to subliminal > supraliminal arousing stimuli in Brodmann Area 13, with a cluster size of 344 voxels mm^2^, ALE value = 2.30.

**Figure 4:**
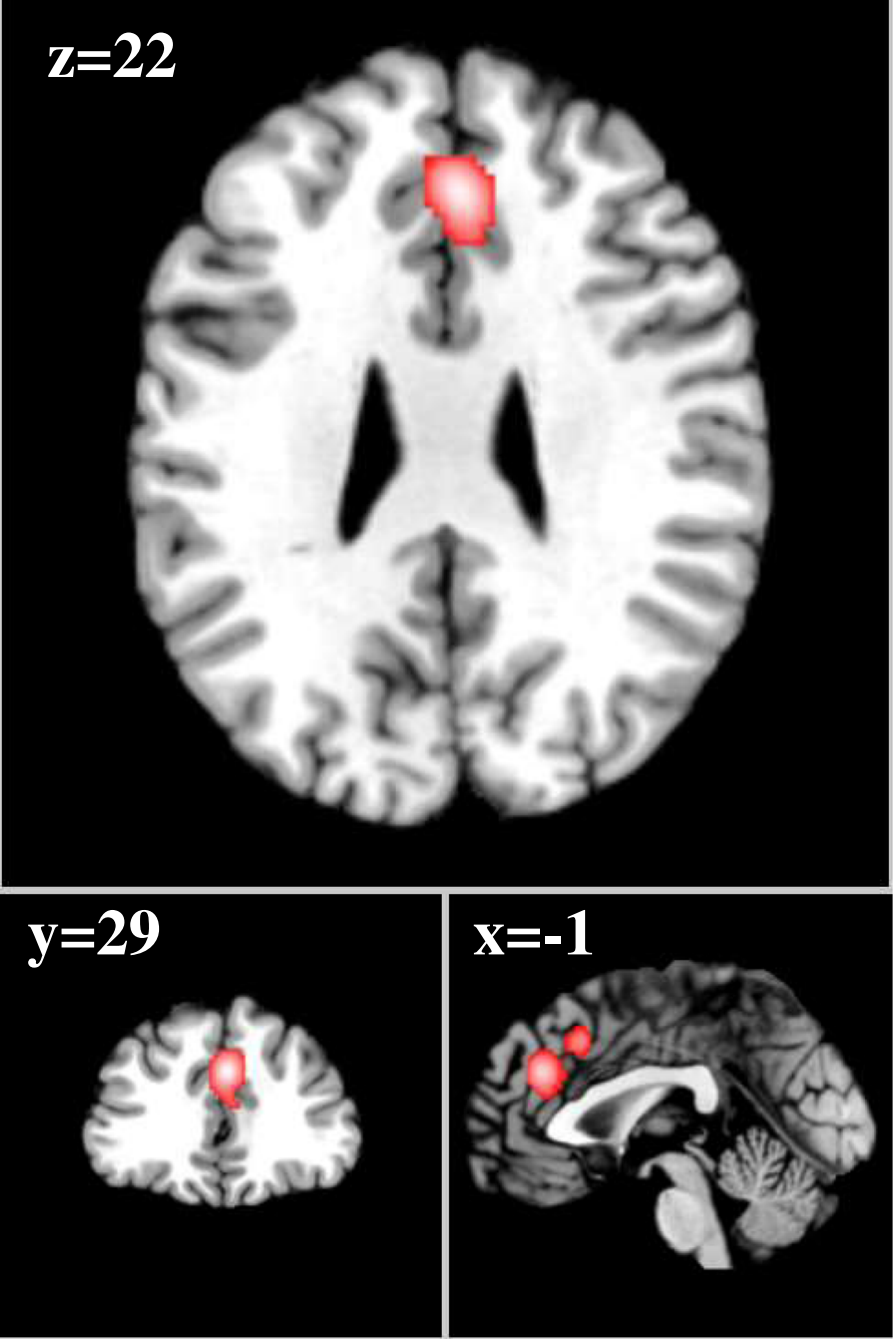
**Caudal anterior cingulate cortex (both subliminal and supraliminal).** Red cluster: subliminal > supraliminal analysis (x = 2, y = 32, z = 36), cluster size of 920 voxels mm^2^, ALE value = 2.52. Green cluster: supraliminal > subliminal analysis (x = 0, y = 19, z = 31), cluster size of 640 voxels mm^2^, ALE value = 4.59. Yellow cluster: area of overlapping. ALE significant activation to both subliminal and supraliminal arousing stimuli in Brodmann Area 32.

See Table 2 and Figures 2, 3 and 4.

**Table 2:** **Results of the ALE analyses, with significantly activated brain regions**

**Cluster** ^**a**^	**Anatomical Label**	**Side**	**Brodmann area**	**Peak voxel coordinates** ^**b**^			**Cluster size (mm** ^**3**^ **)**	**ALE value (×10** ^**−2**^ **)**
				**x**	**y**	**z**		
*Subliminal > Supraliminal*								1008
1	Fusiform Gyrus	Right	19	47	−71	−3	1008	4.12
2	Caudal Anterior Cingulate Cortex	Right	32	2	18	36	920	2.52
3	Insula	Right	13	37	4	−5	344	2.30
*Supraliminal > Subliminal*								4168
1	Anterior Cingulate Cortex	Left	32	−2	34	18	1464	6.20
2	Caudal Anterior Cingulate Cortex	Left	32	0	19	31	640	4.59

^a^ALE clusters threshold at p < 0.05 (cluster-level uncorrected p, corrected for multiple comparisons, cluster-forming threshold at voxel level p <0.001).

^b^Voxel coordinates are in Talairach space.

For a schematic illustration of where these regions are in the brain, and possible connections, see Figure 1.

## Discussion

We present preliminary meta-analyses of fMRI studies that compare the effects of subliminal versus supraliminal presentation of the same stimulus on brain activation. When interpreting these findings, the categories and differences between visual and tactile stimulation must be considered with caution, as they may influence the data observed. Specifically, left anterior cingulate cortex (ACC) was most significantly activated across all studies when supraliminal processing was the reported activation; the right fusiform gyrus/middle occipital gyrus and right insula when subliminal processing was reported, and the caudal anterior cingulate cortex to both levels of perception. Additionally, it appears that subliminal stimulation most often activates regions of the right hemisphere, whereas in contrast, supraliminal stimulation appears to activate the left hemisphere. This is intriguing given that the right hemisphere is typically associated with emotional processing, whereas the left hemisphere is linked to language processing and higher level emotional processing that is largely consciously perceived (Bauer et al. 2014; Shobe 2014). This could suggest that conscious processing is linked to left hemisphere, language-based processing, such as cognitive labeling, whereas the right hemisphere maybe more associated with non-conscious processing of one’s ‘gut-feelings’ and instincts. However, the different stimuli included in these meta-analyses may have influenced the results, and were: auditory tones (although these were under-represented in the final meta-analysis); rectal stimulation; famous, angry and disgusted faces and sexual images. Nevertheless, all of these types of stimuli have in common that they stimulate sensations in the peripheral nervous system.

Our hypotheses, that consciously perceived stimuli activate prefrontal cortex regions, in comparison to unconscious perception of the same stimuli, were partially supported, in that the ACC can be regarded as part of the prefrontal cortex system. However, we did not, as expected, find subcortical regions (e.g. amygdala, hippocampus, striatum) being activated to non-consciously presented stimuli, but instead found that the right fusiform gyrus and right insula cortices were most significantly activated by subliminal stimuli. However, again it must be considered that these observations could be due to the type of stimuli used (e.g. faces and rectal stimulation), rather than as a consequence of variance in conscious perception. Most other fMRI studies using subliminal paradigms compare subliminal arousing to subliminal neutral stimuli, but the studies included in this review only compared supraliminal and subliminal presentation of the same stimulus within the same study (Brooks et al. 2012). The preliminary findings we present here for the first time compare neural activation to conscious and unconscious processing of the same stimulus, either in a direct comparison of subliminal versus supraliminal stimulation, or including subliminal versus - and supraliminal versus neutral contrasts using the same stimulus within the same study. Our meta-analysis lends support to some current theories about the neural correlates of consciousness, and also has the potential to progress our understanding of psychological processes, by providing a priori brain regions involved in the delineation of automatic non-conscious states from conscious experience. Next, we discuss these findings in relation to the different levels of perceptual awareness, and theories of consciousness.

### Unconscious perception of stimuli

The right fusiform gyrus, part of the middle occipital gyrus, was most consistently activated across the fMRI studies included in this review, in response to subliminally presented arousing stimuli that were not consciously perceived. This result could be driven by more studies that employed the presentation of faces in this meta-analysis, although it is nonetheless interesting to observe that non-consciously processed faces activate this region. While the fusiform gyrus is most well-known as the ‘fusiform face area’, particularly during conscious perception of faces, activation in this area may also be associated with non-verbal facial communication (Kreifelts et al. 2013), which is perhaps more implicit on first glance. Furthermore, the middle occipital gyrus is associated with the decoding of affectively arousing stimuli (Dima et al. 2011). It is connected with the amygdala, a brain region associated with unconscious processing (Slipp 2000) and also general arousal (Costafreda et al. 2008) and may be associated with the processing of bottom up stimuli to influence declarative memory.

Another area we found to be significantly activated by subliminal stimulation is the right posterior insula cortex (PIC), which is in agreement with our previous meta-analysis of fMRI studies (Brooks et al. 2012). The insular cortex is traditionally linked to conscious interoceptive awareness and the perception of one’s own body (Craig 2010; Craig 2009). However, given the insula’s connectivity to subcortical and cortical regions, this brain region could also adhere to the role of “director” of somato-sensory responses from the internal mileu, which may pre-empt conscious decisions or awareness. Therefore, it is plausible that the insular cortex would be activated in response to subliminal stimuli in order to modulate a consequential conscious response to a change in somato-sensory or visceral stimulation. The data we present here suggests that a perception of perturbations in the body can occur without conscious awareness, and might be encoded as activation at the level of the primary occipital cortex (perhaps via connections to the amygdala) and the insular cortex.

### Anterior cingulate cortex: a gateway between pre-attentive bottom-up and top-down cognitive evaluation?

Activation of the anterior cingulate cortex (ACC) was observed across studies in response to both subliminal and supraliminal arousing stimuli in this review. This brain area is considered crucial in the detection of error following a false mental prediction and in the detection of internal conflict, such as dissonance between two competing goals, but it is unclear whether this is associated with conscious perception of the stimulation or not (Charles et al. 2013). Some evidence suggests that the greater the conscious processing exerted, the higher the activation that is observed in the ACC (Mulert et al. 2005). Additionally, the insular cortex and ACC have strong connections that elaborate on emotional feelings and play a role in sensory perception and conscious evaluation (Critchley 2005). The IC-ACC network has also been linked to conscious self-recognition (Devue et al. 2007) and is implicated in conscious executive processes (De Pisapia et al. 2011). Prediction error detection is largely associated with activation of the ACC and this is also in line with contemporary views of emotion and the experience of *presence*, purporting that an emotional sense of self is not simply derived from sensing interoceptive signals, but also determined by prediction error processing, or how our belief systems match reality (Seth et al. 2011). This lends support to the view that the ACC functions as a gateway between automatic primary process affective states and higher order cognitive processing, particularly when affect and cognition are in conflict, or in psychiatric conditions such as post-traumatic stress disorder (Botvinick et al. 1999; Cohen et al. 2013). A conflict may also occur in the absence of awareness, when the body’s physiology is unexpected perturbed, as shown for example in this meta-analysis, where some of the included studies used stimuli that altered the physiological state of the body without conscious awareness. However, the different types of stimulation included in this review may have confounded our findings, and so caution must be taken with interpretation. Furthermore, despite the original stimulus being unconsciously perceived, subsequent bodily reflexes, such as heart rate variability, tactile stimulation, perspiration, muscle tension are likely to be consciously perceived (e.g. the basis of a gut feeling). In a recent study, the presentation of subliminal sexual images was linked to ACC activation and potential cognitive conflict in men, as sexual affective states were engaged in the brain, but not indulged, which likely led to a conscious perception of frustration (Gillath & Canterberry 2011). Furthermore, others show that there is a dynamic relationship between bottom-up primary sensory activations and top-down modulation by the ACC (Crottaz-Herbette & Menon 2006), formulating an eventual global, or ‘bigger picture’ perspective. It is likely that affect processing, whether at first consciously perceived or not, alters prefrontal cortical systems via the ACC. Translating this in relation to the stimuli used in the fMRI studies presented here, one might argue that stimulation of the rectum and emotional faces (the most commonly used stimuli in the studies included in this review) all evoke arousal states deep in the brain that perturb pre-attentive neural circuits. The level of ACC involvement in this process, subsequent interoceptive awareness and cognitive evaluation of bodily state in response to an affective stimulus, is likely to be biased by previous experience in line with current self-referential goals and contextual cues.

### Linking our findings to theories of consciousness

Our data were not able to provide direct support for Damasio’s Somatic Marker Hypothesis, which implicates the ventro-medial prefrontal cortex and periaqueductal gray in the influence of non-conscious processes on conscious decision making (Damasio 2010; Damasio 1994). However, the studies presented here did not measure decision making processes. Some contemporary theories of consciousness purport that the experience of 'qualia' or the subjective awareness of one's self perceiving (e.g. what is it like to experience the colour red?), is achieved by attention mechanisms in prefrontal cortical systems, such as the ACC, being directed from 'backstage' signals that are represented by distinct neural signatures in the mesolimbic brain regions, such as the striatum (Baars & Franklin 2003) and primary visual areas for mental imagery. Baars, in his Global Workspace Theory (GWT) proposes the view that unconscious processes, such as those derived from subliminal visual stimuli, interact with cognitive processes in the PFC, such as working memory, to cognitively frame a consciously-perceived self-relevant goal (Baars & Franklin 2003), which may also be referred to as a ‘cognitive bias’. Others support Baars’ global workspace theory, implicating the ACC and areas that connect to this region (e.g. insula cortex, visual cortex, mesolimbic regions), enabling consciousness to be directed by a vast network of backstage processes supporting neural functions that are not consciously perceived, (Dehaene et al. 2006) . Thus, although our meta-analysis highlights brain regions involved in non-conscious sensory (as opposed to cognitive) processing, it could be that activation of the ACC, visual cortex and insula by non-consciously perceived stimuli could further influence downstream prefrontal cortex systems (via the ACC as a gateway to other PFC systems) associated with higher-order cognitions (e.g. working memory).

Other contemporary theories of consciousness focus on how non-conscious processing can influence behavior and prime responses to stimuli (Eimer & Schlaghecken 2003). It has been shown that masked, and thus non-consciously perceived stimuli can alter preferences and speed of choice, which may for example, be the basis of impulsive responses. Response facilitation and inhibition in subliminal priming is suggested to involve fronto-striatal circuits (Eimer & Schlaghecken 2003) and could be a key to understanding triggers for impulsive behaviours in some psychiatric disorders (e.g. addiction, aggression, eating disorders).

However, our data did not implicate fronto-striatal circuitry (only ACC) in unconscious processing, but instead found that non-consciously processed stimuli activate visual cortex, insula and ACC. Against the background of the current data, must proceed with caution when choosing subliminal paradigms to test theories of unconscious perception, given our current lack of knowledge regarding the underlying mechanisms of subliminal stimulation. For example, it is not currently known to what extent the semantic context of masked stimuli is processed at an unconscious level, and whether subliminal stimulation activates processes that linger in the brain for secondary higher-order conscious processing. Given these limitations to our current knowledge, subliminal paradigms may not be the best choice for collecting data on unconscious processes. Nevertheless, subliminal paradigms may be valuable for probing arousal mechanisms in the brain that are independent of cognitive modulation (Brooks & Stein 2014) especially if a direct comparison of subliminal versus supraliminal effects on the brain using the same stimulus is conducted.

### Limitations

We found 9 studies reporting the neural correlates of subliminal activation, and 10 studies reporting the neural correlates of supraliminal activation. Given that our sample size was small, our data must be regarded as preliminary, providing basic insights into the neural correlates of conscious processing that need further clarification with additional brain imaging studies. Additionally, the studies included were heterogeneous with a bias towards stimulation with images of faces and rectal stimulation, which likely drove the findings we obtained. Related to the heterogeneity of studies, we also included both studies that provided a direct comparison between subliminal and supraliminal stimulation, as well as studies that compared subliminal and supraliminal stimulation separately to a neutral condition. We did this so that we could include all studies that examined the same subliminal and supraliminal stimulus in their publication, even if they did not directly compare these levels of stimulation. Also, it must be noted that although the participants in this meta-analysis were psychologically healthy, a small number of participants had existing medical conditions (e.g. GERD, IBS), which may have influenced brain function. Furthermore, the ALE approach we adopted does not take into account the relative strength of activation reported by each study, but the present version is essentially a 'vote-counting' method of reported coordinates weighted for the number of participants per study. There were not enough studies examining separately neural activation in males and females, which, as one of the studies has shown (Gillath & Canterberry 2011), may be important in terms of gauging different levels of cognitive control exerted over arousing stimuli. Furthermore, the stimuli, although commonly activating bodily sensations, were quite diverse, incorporating auditory tones, somatosensory and visual stimulation, and there were not enough studies using one particular modality to conduct separate meta-analyses.

## Conclusions

While our data is preliminary, it suggests that perception of non-consciously perceived stimuli activates anterior cingulate cortex (ACC) and insular cortex, to form a basis for conscious perception. Activation of primary visual areas by non-consciously perceived stimuli is perhaps driven by a bias for these studies to use images of emotional faces, and so more fMRI studies are needed to compare subliminal and supraliminal presentation of other types of stimuli in different modalities. After further fMRI studies comparing the neural correlates of subliminal versus supraliminal stimulation, meaningful conclusions are more likely to be drawn about brain systems involved in unconscious perception.

## Additional files

Additional file 1: Table S1. A list of the excluded studies not included in our meta-analyses. Additional file 2: Table S2. MOOSE Checklist details.
