# Supplementary material for: Subliminal versus supraliminal stimuli activate neural responses in anterior cingulate cortex, fusiform gyrus and insula: a meta-analysis of fMRI studies
Source: BMC Psychol. 2014 Dec 11;2(1):52. doi: 10.1186/s40359-014-0052-1 (PMC4271330; doi:10.1186/s40359-014-0052-1)
Supplement: Additional file 1: Table S1. — A list of the excluded studies not included in our meta-analyses. [file 40359_2014_52_MOESM1_ESM.docx]

**Additional file 1: Table S1.**

| Study (author/year of publication Reason for exclusion | | |
| --- | --- | --- |
| Excluded from review (n=20) | | |
| Artiges et al., (2006)  Brázdil et al., (2001)  Carrubba et al., (2010)  Dehaene et al., (2006)  Dietrich (2008)  Eimer et al., (2003)  Fischmeister (2010)  Gaillard et al., (2006)  Gaillard et al., (2007)  Grosbras et al., (2003)  Henson et al., (2008)  Hsu et al., (2007)  Jaffard et al., (2008)  Kim et al., (2010)  Kjaet et al., (2001)  Litré et al., (2010)  Négrevergne et al., (2003)  Shaker et al., (2007)  Smeets et al., (2011)  Windischberger et al., (2010) | Subliminal data was not reported  Event-related potentials (ERP) not fMRI  Subliminal data not available  No experimental data, theoretical  Review  Review  Not fMRI  1 patient  Review, French  Transcranial Magnetic Stimulation with fMRI  Electro-encephalo-graphy (EEG)  Not sublimnial  Not subliminal  Not subliminal  Positron Emission Tomography (PET)  Article in French  Article in French  Review  Not subliminal  German, review | |
| Excluded from meta-analysis (n=15) ??? | | |
| Blankenburg et al, (2003)  Boy et al., (2010)  D'Ostillo & Garraux (2011)  Dehaene et al., (2003)  Hesselmann et al., (2008)  Hesselmann et al., (2010)  Kern et al., (2002)  Kern et al., (2009)  Lawal et al., (2006)  Morris et al., (2007)  Naccache et al., (2001)  Nakamura et al., (2005)  Nakamura et al., (2007) | No Talairach or MNI coordinates reported  No Talairach or MNI coordinates reported  No Talairach or MNI coordinates reported  No Talairach or MNI coordinates reported  No Talairach or MNI coordinates reported  No Talairach or MNI coordinates reported  No Talairach or MNI coordinates reported  No Talairach or MNI coordinates reported  No Talairach or MNI coordinates reported  No Talairach or MNI coordinates reported  No Talairach or MNI coordinates reported  No Talairach or MNI coordinates reported  No Talairach or MNI coordinates reported | |
| Excluded because of the new critery of selection (n=27) | |  |
| Andersen et al, (2005) | Subliminal contrast with pain |  |
| Bianchi- Demichelli et al, (2009) | No contrast between subliminal and supraliminal |  |
| Dannlowski et al,(2007) | No contrast between subliminal and supraliminal |  |
| Dehane et al, (2004) | No contrast between subliminal and supraliminal |  |
| Degonda et al, (2005) | Correlation Analysis |  |
| Diaz et al, (2007) | No contrast between subliminal and supraliminal |  |
| Duan et al, (2010) | No contrast between subliminal and supraliminal |  |
| Janzen et al, (2007) | No contrast between subliminal and supraliminal |  |
| Killgore et al, (2004) | No contrast between subliminal and supraliminal |  |
| Kern et al., (2003) | ??? |  |
| Koudier et al, (2005) | No contrast between subliminal and supraliminal |  |
| Koudier et al, (2009) | No contrast between subliminal and supraliminal |  |
| Koudier et al, (2010) | No contrast between subliminal and supraliminal |  |
| Lawal et al, (2008) | No contrast between subliminal and supraliminal |  |
| Liddell et al, (2005) | No contrast between subliminal and supraliminal |  |
| Luo et al, (2004) | No contrast between subliminal and supraliminal |  |
| Naccache et al, (2005) | No contrast between subliminal and supraliminal |  |
| Nomura et al, (2004) | No contrast between subliminal and supraliminal |  |
| Ortigue et al, (2003) a and b | No contrast between subliminal and supraliminal |  |
| Pannese et al, (2011) | No contrast between subliminal and supraliminal |  |
| Pessiglione et al, (2007) | No contrast between subliminal and supraliminal |  |
| Pessiglione et al, (2008) | No contrast between subliminal and supraliminal |  |
| Qiau et al, (2010) | No contrast between subliminal and supraliminal |  |
| Smith et al, (2011) | Only liminal contrast with subliminal |  |
| Suslow et al, (2010) | No contrast between subliminal and supraliminal |  |
| Wolbers et al, (2006) | No contrast between subliminal and supraliminal |  |
|  |  |  |
|  |  |  |
|  |  |  |
|  |  |  |
|  |  |  |
|  |  |  |
|  |  |  |
|  |  |  |
|  |  |  |
|  |  |  |
|  |  |  |
|  |  |  |
