# Supplementary material for: Subliminal versus supraliminal stimuli activate neural responses in anterior cingulate cortex, fusiform gyrus and insula: a meta-analysis of fMRI studies
Source: BMC Psychol. 2014 Dec 11;2(1):52. doi: 10.1186/s40359-014-0052-1 (PMC4271330; doi:10.1186/s40359-014-0052-1)
Supplement: Additional file 2: Table S2. — MOOSE Checklist details. [file 40359_2014_52_MOESM2_ESM.doc]

**MOOSE Checklist**

**Subliminal versus supraliminal stimuli activate neural responses in anterior cingulate cortex, fusiform gyrus and insula: a meta-analysis of fMRI studies**

| **Criteria** | | **Brief description of how the criteria were handled in the meta-analysis** |
| --- | --- | --- |
| **Reporting of background should include** | |  |
| √ | Problem definition | Non-conscious processing may underlie various psychological processes. However, the neural substrates of non-conscious processing have not been entirely elucidated. |
| √ | Hypothesis statement | Examining the differential effects of stimuli that are consciously, versus unconsciously perceived will improve our knowledge of neural circuitry involved in non-conscious perception. |
| √ | Description of study outcomes | Here we conduct preliminary analyses of neural activation in studies that have used both subliminal and supraliminal presentation of the same stimuli. |
| √ | Type of exposure or intervention used | The same stimuli consciously and unconsciously perceived |
| √ | Type of study designs used | We use Activation Likelihood Estimation (ALE) to conduct a preliminary examination of functional Magnetic Resonance Imaging (fMRI) studies |
| √ | Study population | Mentally healthy participants |
| **Reporting of search strategy should include** | |  |
| No | Qualifications of searchers |  |
| √ | Search strategy, including time period included in the synthesis and keywords | PubMed, Medline, Ovid, Sciencedirect, Web of Science and Google Scholar were searched, and hand searches of reference lists up to October 2013; keywords: subliminal and supraliminal stimulation |
| √ | Databases and registries searched | PubMed Medline, Ovid, Sciencedirect, Web of Science and Google Scholar |
| √ | Search software used, name and version, including special features | We didn’t use a software for research. |
| √ | Use of hand searching | We hand-searched bibliographies of retrieved papers for additional references, |
| √ | List of citations located and those excluded, including justifications | Details of the literature search process are outlined in the text. |
| √ | Method of addressing articles published in languages other than English | We used only English articles |
| √ | Method of handling abstracts and unpublished studies | We didn’t use unpublished studies |
| √ | Description of any contact with authors | We didn’t contact any authors |
| **Reporting of methods should include** | |  |
| √ | Description of relevance or appropriateness of studies assembled for assessing the hypothesis to be tested | Detailed inclusion and exclusion criteria were described in the methods section. |
| √ | Rationale for the selection and coding of data | Data extracted from each of the studies were obtained by a subliminal or supraliminal stimulation. |
| √ | Assessment of confounding | We checked all studies by mental health of subjects. |
| √ | Assessment of study quality, including blinding of quality assessors; stratification or regression on possible predictors of study results | Not applicable |
| √ | Assessment of heterogeneity | We selected studies with different kind of stimuli without any limitation |
| √ | Description of statistical methods in sufficient detail to be replicated | Description of ALE methods |
| √ | Provision of appropriate tables and graphics | We included 2 tables and 4 figures |
| **Reporting of results should include** | |  |
| √ | Graph summarizing individual study estimates and overall estimate | Table 1 |
| √ | Table giving descriptive information for each study included | Table 1 |
| √ | Results of sensitivity testing | Table 2 |
| √ | Indication of statistical uncertainty of findings | ALE clusters threshold at p<0.05 (cluster-level uncorrected p, corrected for multiple comparisons, cluster-forming threshold at voxel level p < 0.001) |
| **Reporting of discussion should include** | |  |
| √ | Quantitative assessment of bias | We found 9 studies reporting the neural correlates of subliminal activation, and 10 studies reporting the neural correlates of supraliminal activation. Given that our sample size was small, our data must be regarded as preliminary, providing basic insights into the brain regions involved in conscious processing |
| √ | Justification for exclusion | See Selected studies |
| √ | Assessment of quality of included studies | Table 1 |
| **Reporting of conclusions should include** | |  |
| √ | Consideration of alternative explanations for observed results | Given that our sample size was small, our data must be regarded as preliminary, providing basic insights into the brain regions involved in conscious processing, that need further clarification with additional brain imaging studies. Additionally, the studies included were heterogeneous, with a bias towards stimulation with images of faces and rectal stimulation, which likely drove the findings we obtained. |
| √ | Generalization of the conclusions | Our results suggest that perception of non-consciously perceived stimuli likely activates anterior cingulate cortex (ACC) and processing via the insular cortex that forms a basis for conscious perception. |
| √ | Guidelines for future research | More fMRI studies are needed to compare subliminal and supraliminal presentation of other types of stimuli in different modalities, so that meaningful conclusions can be drawn about brain systems involved in unconscious perception |
| √ | Disclosure of funding source | This work was supported by the Brain Behaviour Initiative, Cape Town, and the Claude Leon Foundation, South Africa., and the Swedish Research Council. |
